# Is Lateropulsion Really Related with a Specific Lesion of the Brain?

**DOI:** 10.3390/brainsci11030354

**Published:** 2021-03-10

**Authors:** Kyoung Bo Lee, Sang Won Yoo, Eun Kyu Ji, Woo Seop Hwang, Yeun Jie Yoo, Mi-Jeong Yoon, Bo Young Hong, Seong Hoon Lim

**Affiliations:** Department of Rehabilitation Medicine, St. Vincent’s Hospital, College of Medicine, The Catholic University of Korea, Seoul 06591, Korea; kblee0732@naver.com (K.B.L.); Yoo7864@gmail.com (S.W.Y.); eunshil7@naver.com (E.K.J.); Hws0609@hanmail.net (W.S.H.); nugry@naver.com (Y.J.Y.); allogen@naver.com (M.-J.Y.)

**Keywords:** stroke, pusher syndrome, lateropulsion, voxel-based lesion symptom mapping, scale for contraversive pushing, postural control, VLSM

## Abstract

Lateropulsion (pusher syndrome) is an important barrier to standing and gait after stroke. Although several studies have attempted to elucidate the relationship between brain lesions and lateropulsion, the effects of specific brain lesions on the development of lateropulsion remain unclear. Thus, the present study investigated the effects of stroke lesion location and size on lateropulsion in right hemisphere stroke patients. The present retrospective cross-sectional observational study assessed 50 right hemisphere stroke patients. Lateropulsion was diagnosed and evaluated using the Scale for Contraversive Pushing (SCP). Voxel-based lesion symptom mapping (VLSM) analysis with 3T-MRI was used to identify the culprit lesion for SCP. We also performed VLSM controlling for lesion volume as a nuisance covariate, in a multivariate model that also controlled for other factors contributing to pusher behavior. VLSM, combined with statistical non-parametric mapping (SnPM), identified the specific region with SCP. Lesion size was associated with lateropulsion. The precentral gyrus, postcentral gyrus, inferior frontal gyrus, insula and subgyral parietal lobe of the right hemisphere seemed to be associated with the lateropulsion; however, after adjusting for lesion volume as a nuisance covariate, no lesion areas were associated with the SCP scores. The size of the right hemisphere lesion was the only factor most strongly associated with lateropulsion in patients with stroke. These results may be useful for planning rehabilitation strategies of restoring vertical posture and understanding the pathophysiology of lateropulsion in stroke patients.

## 1. Introduction

Lateropulsion (pusher syndrome) is a postural bias toward the weak side [1,2] accompanied by resistance to postural correction back to a vertical upright position after stroke [3,4,5]. Patients with pusher syndrome are able to consciously perceive body orientation, and are capable of spontaneous postural adjustment of the non-paretic leg in the roll plane [6]. Clinically, lateropulsion may be a barrier to sitting, standing or gait after stroke. Thus, a better understanding of lateropulsion would be beneficial for treatment and restoration of vertical postural control.

Previous research has suggested that the right hemisphere plays a key role in an “internal model of verticality”, and in the control of body orientation with respect to gravity [5]. In addition, thalamoparietal projections have a functional role in the processing of somasthetic graviceptive information [5]. Research has also shown that the inferior parietal lobe plays a role in lateropulsion [3]. Other reports have demonstrated that injury to the insular cortex, postcentral gyrus and posterior thalamus might be associated with lateropulsion [7].

Taken together, previous research suggests that the right hemisphere may play a role in vertical postural control. However, the areas in the right hemisphere underlying lateropulsion have yet to be clearly identified. Therefore, the present study aimed to determine the specific right hemisphere brain lesions responsible for lateropulsion using voxel-based lesion symptom mapping (VLSM) methods, including analyses of 3T brain magnetic resonance imaging (MRI) scans, and clinical evaluations of patients who had experienced supratentorial strokes for the first time.

## 2. Materials and Methods

### 2.1. Study Design and Participants

This retrospective cross-sectional study included the data of 50 right handed patients who had experienced a supratentorial right hemisphere stroke for the first time, as confirmed by assessment of neurologic symptoms and initial brain imaging studies such as MRI or computed tomography (CT). All patients were recruited from a single inpatient/outpatient center from May 2015 to April 2018, and all had suffered supratentorial strokes and were undergoing inpatient rehabilitation therapy at our institution. The inclusion criteria were as follows: (1) aged >20 and ≤80 years; (2) first unilateral stroke lesion in the right hemisphere; (3) ability to follow verbal instructions; and (4) initial assessment within 2 weeks of onset [8]. The exclusion criteria were as follows: (1) missing medical records or 3T brain MRI scans; (2) any symptomatic brain disorders other than stroke; and (3) any other medical disorder resulting in problems with independent activities of daily living (ADL) or balance. The diagnosis of lateropulsion was confirmed at the initial assessment (within 14 days after stroke onset) using the Scale for Contraversive Pushing (SCP); [9]. The rehabilitation program was initiated within 7 days of stroke onset in all subjects, and continued for up to 6 months, which consisted of physical and occupational therapies based on a neurodevelopmental treatment approach (physical therapy) and a task-orientated approach (occupational therapy). The program consisted of 1–2 h sessions (5 days per week) that included both physical and occupational therapy components [10]; subjects also received speech therapy as needed. All interventions were primarily focused on using and strengthening the affected limb, basic mat activity, symmetric weight bearing and transfer activities and gait training, and were not performed exclusively for a specific purpose [11].

The study protocol was reviewed and approved by the Institutional Review Board of Catholic University, College of Medicine (Registry No. VC18RESI0109); the requirement for informed consent was waived by the board.

### 2.2. Clinical Assessments

The demographic data of all subjects were retrospectively reviewed. Contraversive pushing was assessed as the primary outcome using standardized SCP [9]. Based on Davies’ criteria [2], SCP assesses (i) symmetry of spontaneous posture while sitting and standing, (ii) use of the arm and/or the leg to increase the area in physical contact with the ground while sitting and standing, and (iii) resistance to passive correction of posture while sitting and standing. Patients with a score greater than zero on all three components were considered to have contraversive pushing. SCP includes three components: (1) symmetry of spontaneous body posture (0, 0.25, 0.75 or 1 point); (2) use of non-paretic extremities (0, 0.5 or 1 point); and (3) resistance to passive correction of the tilted posture (0 or 1 point) [9,12]. Each component is tested in both sitting and standing positions, yielding a maximum score of 2 per component. Karnath et al. [9] originally recommended a diagnostic cutoff score of ≥1 for each component (sitting and standing). The present study employed a less conservative cutoff of >0 for each component, as stated above, at the initial assessment after onset. This is in accordance with Baccini et al. [4,13], who reported improved diagnostic accuracy with this cutoff.

The Motricity Index is a brief instrument for assessing motor impairment, by examining the upper extremity (for pinch grip strength, elbow flexion and shoulder abduction) and the lower extremity (for hip flexion, knee extension and ankle dorsiflexion) [14,15]. The Motricity Index involves one movement each for the upper and lower extremities, rated according to a 6-point scale and yielding a maximum score of 33 for each joint or location. The values are summed across joints; a score of 100 indicates the absence of any impairment. Functional disability was assessed using the Korean version of the Modified Barthel Index (MBI) [16,17]. Patients were classified as having spatial neglect when they showed typical clinical behavior such as (i) spontaneous deviation of the head and eyes toward the ipsilesional side, (ii) orienting toward the ipsilesional side when addressed from the front or the left, and (iii) ignoring of contralesionally located people or objects [18,19]. Balance ability was tested using the Berg Balance Scale (BBS), which includes 14 functional balance items. Scores range from 0 to 56 points; better balance is indicated by a higher score [20,21,22]. Cognitive function was evaluated using the Mini Mental State Examination (MMSE) [23].

### 2.3. Lesion Analysis

The time between MRI data obtained for lesion analysis and stroke onset was 2.97 ± 6.15 days. Lesions were mapped using MRIcron software (available online: https://www.nitrc.org/projects/mricron). A trained image analyst manually highlighted lesions on individual 3T MRI scans; these were then confirmed by an experienced clinical psychiatrist blind to all clinical data [8]. For more accurate analyses, the origin of each image (coordinates: 0 × 0 × 0 mm^3^) was reoriented such that it was located close to the anterior commissure. To analyze the lesion maps, segmentation and normalization were employed [8,11,24]. We used the MR segment-normalize and MR normalize functions of a plugin (available online: https://www.nitrc.org/projects/clinicaltbx/) for mapping in stereotaxic space using the normalization algorithm provided by SPM8 software (available online: http://www.fil.ion.ucl.ac.uk/spm/software/spm8). Flair or diffusion weighted imaging (DWI) images were coregistered with each participant’s T1 or T2 MRI, and the T1 and T2 lesion maps were then normalized to Montreal Neurologic Institute (MNI) space using statistical parametric mapping (SPM). Unified segmentation normalization was performed on the anatomical scan [25]. Normalized lesions were subjected to statistical mapping analysis using VLSM algorithms implemented with the statistical nonparametric mapping (SnPM) toolbox (available online: https://warwick.ac.uk/fac/sci/statistics/staff/academic-research/nichols/software/snpm) [26]. A voxel-based lesion symptom mapping (VLSM) procedure was developed to analyze the relationship between tissue damage and behavior on a voxel-by-voxel basis. VLSM analysis identified clusters of voxels with statistically significant t values comparing voxel-wise clinical measurement (SCP) with lesions to those without lesions. Statistical significance was determined following a permutation correction at *p* < 0.05 familywise error correction for comparisons. In addition, to control the other contributors to pusher behavior, we also performed VLSM controlling for lesion volume as nuisance covariates in the multivariate model [26]. We also performed VLSM controlling for other factors potentially contributing to pusher behavior, such as lesion volume [26]. All voxels defining the lesion (1 voxel = 1 mm^3^) were counted using MRIcron software. An overlap map was then generated by summing all of the maps to identify common regions affected by lesions among the stroke population. Colored lesions on the MNI template brain were labeled according to anatomical region and Brodmann area using the xjView 8 toolbox (available online: https://www.nitrc.org/projects/xjview for SPM 8 software).

## 3. Statistical Analyses

Differences between the groups with and without lateropulsion, in terms of demographic data, were assessed using the *t*-test and chi-squared test, as appropriate. Data are expressed as mean values for continuous variables, and as frequencies and percentages for categorical variables. All data were analyzed using IBM SPSS Statistics (version 21.0; IBM Corp., Armonk, NY, USA). Spearman’s correlation analyses were used to assess the associations of pusher behavior with various clinical measures. Stepwise multivariate regression analysis was then performed to further explore the association of significant variables in the correlation analysis with SCP. In the regression model, if the association between SCP and another variable was significant according to Spearman’s correlation coefficient, it was considered a confounder with respect to the SCP value. The following variables were entered as independent variables into the regression model: lesion volume, MBI, MMSE, interval between stroke onset and baseline assessments, hospitalization days, stroke type, strength of upper and lower limbs, BBS and the presence of spatial neglect. The level of significance was set at *p* < 0.05.

## 4. Results

The clinical and demographic characteristics of all subjects are demonstrated in Table 1. There were no significant differences in the two groups for age, gender and the presence of dysarthria. However, significant differences between groups were founded for lesion volume, stroke type, the interval between stroke onset and baseline assessment, hospitalization periods, the presence of neglect, MMSE, MBI, motricity upper extremity (UE) and lower extremity (LE) and SCP.

Correlation analysis between SCP and other variables was conducted, and the differences between groups were investigated and included in the difference of the independent variables. In the regression analyses of factors related to SCP, lesion volume was only identified as the best contributor of SCP and summarized in Table 2.

For each region, the Montreal Neurological Institute (MNI) coordinates of the center of mass are provided, and were obtained using statistical non-parametric mapping (SnPM). Each voxel cluster survived the threshold of *p* < 0.05. FWE is familywise error corrected.

**Figure 1:**
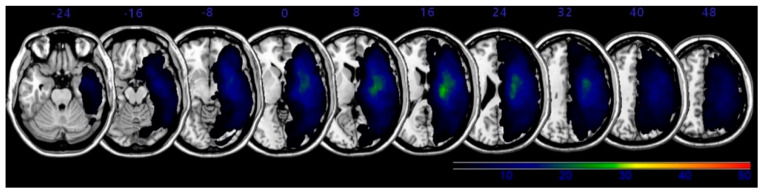
Lesion overlap map for all subjects (*n* = 50). The map of overlapping lesion areas in the brains of all stroke patients was created in standard Montreal Neurologic Institute (MNI) space. Colors denote the frequency of overlap.

**Figure 2:**
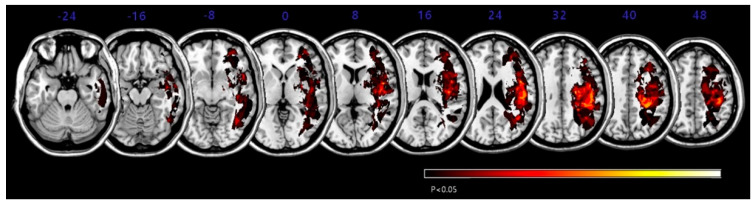
Voxel-based lesion symptom mapping (VLSM) was combined with statistical non-parametric mapping (SnPM) to associations of lesion areas with Scale for Contraversive Pushing (SCP) scores. Lesion overlay map in Montreal Neurologic Institute (MNI) space for SCP scores. Only voxels significant at *p* < 0.05 (familywise error correction) are shown in red to yellow color. Numbers are the z-coordinates in MNI space; the patient’s right hemisphere is on the right side of the figure. After adjusting for lesion volume as a nuisance covariate, no lesion areas were definitively associated with SCP.

The overlapping lesions in the brains of all stroke patients were created on a standard MNI space brain. (Figure 1; the color represents the frequency of overlap). The VLSM method with SnPM (statistical non-parametric mapping) demonstrated the region that corresponded to SCP (Figure 2 and Table 3). Before adjusting the lesion volume, the precentral gyrus, postcentral gyrus, inferior frontal gyrus, insula and subgyral parietal lobe of the right hemisphere were noted for producing the lateropulsion. However, after adjusting the lesion volume as nuisance covariates, no statistically defined lesion areas were associated with SCP scores in Table 3.

## 5. Discussion

Previous studies suggested that disruption of networks in the right hemisphere could impair vertical postural control and lead to lateropulsion [5]. The postcentral gyrus and surrounding white matter, and the postcentral gyrus and Brodmann area 40 in the inferior parietal lobe, were reportedly associated with lateropulsion [3,7,27]. Damage to the inferior frontal gyrus and precentral gyrus was previously reported to be associated with lateropulsion [3,27,28]. The insula may play a role in vestibular function [29], and damage in this area might disrupt the vestibulospinal system and induce lateropulsion. The precentral gyrus, postcentral gyrus, subgyral parietal lobe, inferior frontal gyrus and insula seemed to be associated with lateropulsion in our preliminary VLSM results. However, with adjusting for the lesion size, there was revealed no definite lesion for lateropulsion. Several reports demonstrated that the size of the lesion was associated with lateropulsion [3,5,7]. Our results indicate that only the larger stroke lesion of the right hemisphere may undermine vertical postural control, regardless of the location of the lesion.

Because the present study was not a large-scale investigation, several statistical methods were used to overcome bias. We analyzed lesions with VLSM, controlling for lesion volume as a nuisance covariate in the multivariate model, and used an SCP score of greater than zero as the diagnostic cutoff. Moreover, we only enrolled patients with lesions in the right hemisphere. However, the limitations of a small sample size and cross-sectional design should be noted. Thus, a large-scale longitudinal study is needed to address the remaining questions and determine the reasons for the small differences in results between previous studies and ours.

The present study demonstrated that the size of the lesion of the right hemisphere would play a role in the development of lateropulsion (pusher syndrome) in patients with stroke.

## 6. Conclusions

Large lesions in the right hemisphere may impair vertical postural control and lead to lateropulsion in patients with stroke. These results may be useful for planning rehabilitation strategies and understanding the pathophysiology of lateropulsion in stroke patients.

## Figures and Tables

**Table 1:** Demographic and clinical data of all subjects.

Characteristics	All Subjects (*n* = 50)	With Lateropulsion	Without Lateropulsion	*p*-Value
		(*n* = 17)	(*n* = 33)	
Age, year (range)	69.08 ± 13.18	69.21 ± 11.42 (47–86)	68.82 ± 16.45 (22–90)	0.923
Gender, *n* (%)				1
Male	28 (56.0)	10 (58.8)	18 (54.5)	
Female	22 (44.0)	7 (41.2)	15 (45.5)	
Stroke type, *n*				
Ischemic/hemorrhagic	36/14	8/9	27/6	0.047
Neglect, *n*	24	8	16	<0.001
Lesion volume voxels (*n*)	61,178.72 ± 82,266.53	127,064.29 ± 100,433.44	27,237.67 ± 42,727.35	0.001
Dysarthria, *n*	2	2	0	0.111
Interval between stroke onset and baseline assessment, days	82 ± 6.52	10.59 ± 7.57	6.39 ± 5.51	0.03
Hospitalization, days	23.00 ± 15.25	31.82 ± 17.47	18.45 ± 11.86	0.009
Mini Mental Status Examination	18.70 ± 7.15	15.00 ± 6.37	20.61 ± 6.86	0.007
Modified Barthel Index	37.26 ± 28.25	17.88 ± 23.04	47.24 ± 25.57	<0.001
Berg Balance Scale	13.20 ± 16.81	1.35 ± 1.58	19.30 ± 17.84	<0.001
Motricity, upper extremity	48.52 ± 22.14	31.71 ± 7.39	57.18 ± 22.27	<0.001
Motricity, lower extremity	51.56 ± 20.14	36.18 ± 8.64	59.48 ± 19.83	<0.001
Scale for Contraversive Pushing	1.71 ± 2.49	5.02 ± 1.16	0.00 ± 0.00	<0.001

**Table 2:** Linear regression analysis of factors associated with Scale for Contraversive Pushing (SCP) scores.

Dependent Variable	Predictors	95% CI	β	*p*
Scale for Contraversive Pushing	Lesion volume	1.479, 1.920	0.342	0.023
	Modified Barthel Index	−0.022, 0.040	0.103	0.555
	Mini Mental Status Examination	−0.175, 0.038	−0.196	0.201
	Interval between stroke onset and baseline assessments (day)	−0.099, 0.127	0.037	0.805
	Hospitalization (day)	−0.030, 0.059	0.087	0.522
	Stroke type	−1.039, 1.718	0.062	0.621
	Motricity—upper extremity	−0.075, 0.037	−0.172	0.489
	Motricity—lower extremity	−0.059, 0.068	0.038	0.883
	Berg Balance Scale	−0.055, 0.057	0.008	0.968
	Neglect	−0.462, 3.316	0.289	0.135

Note: CI%, confidence interval; β, standardized regression coefficient.

**Table 3:** MNI coordinates of brain lesions associated with lateropulsion.

Measures	MNI Coordinates (X, Y, Z)	Voxel Level, *p FWE*	Anatomical Brain Lesion
Scale for Contraversive Pushing (SCP)	48, 0, 7	0.0156	Precentral gyrus (Brodmann area 44)
	53, −21, 20	0.0080	Postcentral gyrus (Brodmann area 40)
	37, −33, 32	0.0080	Subgyral parietal lobe (Brodmann area 40)
	49, 0, 21	0.0064	Inferior frontal gyrus (Brodmann area 9)
	41, −13, 13	0.0186	Insula (Brodmann area 13)
	28, −34, 36	0.0074	Precentral gyrus
SCP controlling for lesion volume	No defined area was detected		

## Data Availability

The data presented in this study are available on request from the corresponding author.
